# Prodrugs in Oncology: Bioactivation and Impact on Therapeutic Efficacy and Toxicity

**DOI:** 10.3390/ijms26030988

**Published:** 2025-01-24

**Authors:** Ritika Kurian, Hongbing Wang

**Affiliations:** Department of Pharmaceutical Sciences, University of Maryland School of Pharmacy, 20 Penn Street, Baltimore, MD 21201, USA; ritika.kurian@umaryland.edu

**Keywords:** prodrug, CYP P450, bioactivation, chemotherapy, active metabolite, pharmacokinetics, cytotoxicity, alkylation

## Abstract

A prodrug is a molecule that lacks pharmacological activity, but upon enzymatic bioactivation, it can generate a therapeutically active molecule. The primary reason behind the design of a prodrug is to help circumvent challenges associated with the physicochemical properties of a drug molecule, such as solubility, absorption, distribution, and instability. Chemotherapy has been at the forefront of cancer treatment for over 70 years due to its ability to target rapidly proliferating tumor cells. However, a major concern with conventional chemotherapy is the lack of selectivity and its associated side toxicity, which can severely impact patients’ quality of life. In oncology, prodrugs have been explored to enhance the bioavailability, improve efficacy, and minimize systemic toxicity of chemotherapeutic agents. Prodrugs activated by enzymes unique to a tumor microenvironment can significantly increase targeted delivery of chemotherapeutic drugs. This review aims to highlight commonly used chemotherapeutic prodrugs, including both alkylating and non-alkylating agents, and discuss their clinical relevance, mechanisms of bioactivation, and toxicity concerns.

## 1. Introduction

The concept of prodrugs was first introduced in 1951 by Dr. Adrien Albert; he described a prodrug as a molecule lacking biological activity but capable of generating a biologically active drug following metabolism [1]. According to this definition and the one accepted by the International Union of Pure and Applied Chemistry (IUPAC), a prodrug is defined as a compound that exerts its pharmacological effects following its biotransformation process [2]. Elegant in design, prodrugs are biologically inert compounds that are activated post-administration to their pharmacologically active forms. Over the years, prodrug design has been established as an effective strategy to improve the physicochemical, biopharmaceutical, or pharmacokinetic properties of an active pharmaceutical ingredient in drug discovery and development.

Currently, prodrugs constitute approximately 10% of all commercially available medicines worldwide. An updated therapeutic target database includes 534 prodrug-drug pairs for 121 targets, of which 146 prodrugs have been approved, and 79 candidates are undergoing clinical trials [3]. Prodrugs can be classified based on their structure, as well as on their site of conversion. They can help improve aqueous solubility, allowing for parenteral delivery. Ionizable promoieties such as succinic acid, amino, or phosphate group enhance solubility by several orders of magnitude. Prodrugs can also improve the solubility of the parent drug at the absorption site, enhancing bioavailability. As ionized prodrugs have poor passive permeability, the amount of intact prodrug entering systemic circulation is minimal, which can reduce the potential for off-target effects.

**Figure 1:**
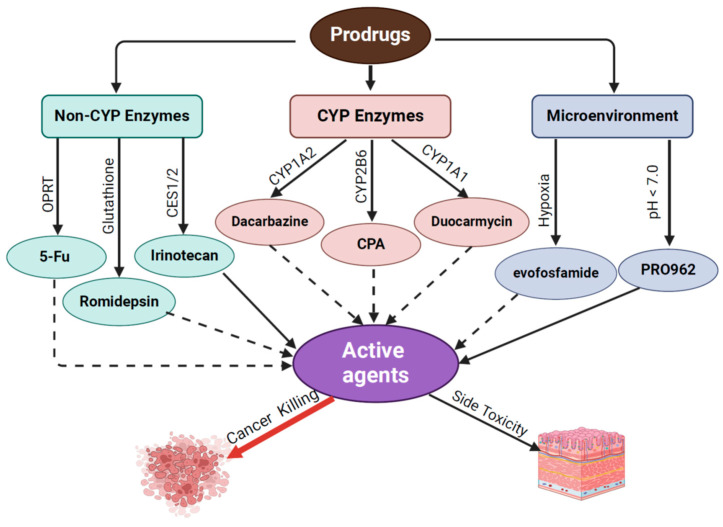
Schematic illustration of chemotherapeutic prodrugs. The figure outlines enzymatic (CYP and non-CYP enzymes)-based and microenvironment (hypoxia and pH)-based bioactivation of prodrugs. The dotted arrow indicates multiple steps involved in the generation of the active agents after the initial metabolism. 5-Fu: 5-Fluorouracil; CPA: cyclophosphamide. The figure was generated using BioRender.

Chemotherapeutic prodrugs are typically formulated via enzymatic or environmental processing to circumvent challenges associated with molecules that lack optimum pharmacological activities (Figure 1). In the case of cancer treatment, chlormethine, also known as mustine, represents the first alkylating chemotherapeutic prodrug [4], although it is no longer commonly used due to severe side toxicity. In 1959, another alkylating agent within the nitrogen mustard family of drugs, cyclophosphamide (CPA), was approved in the U.S. for cancer treatment. Initial hypothesis suggested that CPA was activated by a phosphoramidase-catalyzed hydrolysis reaction occurring inside cancer cells. However, it was observed that tumor cells sensitive to CPA in vivo were longer responsive to it in vitro, indicating that another primary mode of CPA bioactivation might be crucial [5]. In 1963, Brock and Hohorst proposed that the “activation of CPA” that occurred in the liver was mediated through the mixed-function oxidase system [6]. This concept was further confirmed by Cohen et al., who also determined that the enzymes involved with the biotransformation of CPA are primarily located in the microsomes [7]. Dacarbazine, another alkylating prodrug, was approved by the FDA for melanoma treatment in 1975, although it was first synthesized by Dr. Y. Fulmer Shealy in 1962 [8]. Subsequently, newer chemotherapeutic approaches began to emerge in clinics, with compounds that mimicked the structure of physiological molecules such as pyrimidines. One such pyrimidine analog, 5-Fluorouracil, played a major role in the treatment of gastrointestinal carcinomas [9]. A further boost to chemotherapeutic treatment occurred with the discovery of antimitotic agents that consisted of topoisomerase inhibitors such as irinotecan [10].

The tumor microenvironment forms a comprehensive ecosystem that is pivotal for cancer cell growth and invasion. Thus, strategies have been extended to design chemotherapeutic prodrugs that can favorably target specific tumor types based on the tumor microenvironment. Various conditions within the tumor microenvironment, such as changes in pH, oxygen, and glutathione levels, can be leveraged to activate prodrugs. Recently, researchers have developed a tumor microenvironment-responsive prodrug for the treatment of triple-negative breast cancer (TNBC) [11]. This prodrug includes doxorubicin conjugated to a neuropeptide, designed in a way where it remains as nanoparticles at physiological pH while transforming into cytotoxic monomers in the acidic tumor microenvironment. A paclitaxel prodrug was also developed to target folate receptors, which are highly expressed in several cancers. This prodrug achieved prolonged blood circulation and enhanced drug accumulation in tumors, with minimal side effects compared to the free form of paclitaxel [12]. Moreover, the selectivity of chemotherapeutic agents can be improved by synthesizing prodrugs based on differential enzyme expression in tumors. For example, a β-gal-based doxorubicin prodrug significantly inhibited colorectal tumor growth compared to free-doxorubicin treatment, with minimal toxicities in a xenograft model [13].

Over the years, there has been a steady increase in the number of prodrugs being developed. From 2008 to 2024, approximately 50 new prodrugs were approved by the FDA, constituting 14% of all new drugs [14]. Recently, prodrug development has also been revolutionized with the advent of antibody–drug conjugates (ADCs) [15,16]. With conventional chemotherapy being riddled with several shortcomings, an alternative prodrug strategy can help mitigate some of these concerns. In this review, we attempt to highlight the different prodrugs utilized for cancer treatment, their clinical significance and mechanism of bioactivation, and discuss the toxicity associated with their use.

## 2. Alkylating Chemotherapeutic Prodrugs

Alkylating agents are anticancer drugs commonly utilized for chemotherapy. Alkylating agents react with electron-rich biological molecules such as DNA and form covalent bonds. Nitrogen mustards were among the first effective anticancer agents, which continue to be prevalent today [17,18]. Bischloroethyl is the characteristic chemical component in all nitrogen mustards, which reacts through the formation of an aziridinium intermediate [19]. The N-7 position of guanosine is alkylated by this aziridinium ion, which causes irreversible interstrand cross-links, ultimately leading to double-stranded DNA breaks that impair the ability of tumor cells to replicate. Most alkylating drugs target DNA bases through mononuclear nucleophilic substitution (S_N_1) or bimolecular nucleophilic substitution (S_N_2). Numerous lesions, ranging from large helix distortions to small base adducts, occur based on the type of alkyl groups and the position of the bases during the reaction [20]. These lesions are highly cytotoxic, targeting rapidly proliferating cancer cells. As summarized in Table 1, we will elaborate on the clinical application, bioactivation scheme, and side effects of alkylating chemotherapeutic prodrugs such as CPA, dacarbazine, duocarmycin, and evofosfamide in this section.

### 2.1. Cyclophosphamide (CPA)

CPA belongs to a class of bi-functional alkylating agents called oxazaphosphorines with antineoplastic and immunosuppressant properties. Metabolism of CPA, generating nitrogen mustard, has a broad indication in cancer. CPA was originally approved in 1959 for the management of malignant diseases [45]. Although functional as a single agent, CPA is typically used in combination with other antineoplastic agents to maximize effectiveness [45]. Additionally, CPA has been included as part of mobilization and conditioning regimens for blood and bone marrow transplantations.

#### 2.1.1. Clinical Significance

CPA has substantial clinical value due to its widespread use for an array of conditions, including myeloproliferative and lymphoproliferative disorders, solid tumors, and autoimmune disorders. Non-Hodgkin lymphoma is a common form of blood cancer, accounting for 4% of cancers in the United States [46]. CPA has been a mainstay for several chemotherapeutic regimens in treating lymphomas, including the aggressive non-Hodgkin lymphoma such as Burkitt lymphoma [21,47]. In the case of Burkitt lymphoma, CPA appears to be particularly effective; a single course of CPA has achieved a durable complete remission [48,49]. However, CPA-based regimens, which include rituximab, CPA, doxorubicin, vincristine, and prednisone (R-CHOP), have only achieved cure rates of 30–40% in other types of non-Hodgkin lymphoma patients [50]. Recently, a phase II study demonstrated a favorable safety and efficacy profile upon intravenous CPA administration to patients with relapsed or refractory B-cell non-Hodgkin lymphoma [25]. Typically, treatment plans for multiple myeloma patients include immunomodulatory drugs such as lenalidomide and proteosome inhibitors such as bortezomib [51,52]. Patients failing standard multiple-myeloma treatments are often alkylating agent-naïve because these agents are generally avoided due to their impairment of hematopoietic stem cell mobilization. This observation prompted Rivell et al. to assess the effectiveness and safety of high-dose CPA for refractory myeloma patients as a salvage therapy [53]. Interestingly, despite a higher proportion of patients with stage 3 disease, improved responses to high-dose CPA were achieved, suggesting that this chemotherapy could be transiently effective in patients refractory to biological agents. Another clinical trial investigating the effectiveness of CPA in chronic lymphocytic leukemia patients with an immunoglobulin heavy-chain variable gene mutation observed a favorable long-term survival rate following primary treatment with a CPA-based regimen including rituximab [54].

Breast cancer is the most common cancer occurring in women in the United States, accounting for 30% of all new female cancers each year [55]. Although breast cancer treatment typically begins with surgery, patients quite often undergo different therapies such as radiation, chemotherapy, and hormone therapy. Chemotherapy is generally administered prior to surgery because it often shrinks the cancer, which facilitates its removal, controls its spread to other tissues, and lowers the incidence of recurrence [56]. Chemotherapy given before surgery is termed neoadjuvant chemotherapy; chemotherapy is referred to as adjuvant when given after surgery [57]. A neoadjuvant or adjuvant chemotherapy regimen usually consists of a combination of drugs given in a specific number of cycles over a defined time frame. Endocrine therapy has been commonly employed to treat breast cancer among elderly patients; however, the overall therapeutic effect is poor [58,59]. Hence, there is a constant need to improve treatment options for this group of patients. Recently, a treatment strategy based on low-dose CPA administration in combination with endocrine therapy resulted in a favorable outcome among elderly patients [22]. Patients attained a high clinical control rate, an objective used to measure the response to this treatment, as well as a progression-free survival of 13 months, indicating a beneficial therapeutic outcome and a crucial immunomodulatory role of CPA. These results are encouraging because, currently, there is an ongoing phase II clinical trial using CPA-based regimens in women over 65 years of age with stage I-III HER2-negative breast cancer [26]. Results from this study could provide further evidence supporting the effectiveness of CPA-based treatment for breast cancer among older vulnerable women. Lung cancer, another prevalent type of cancer, with around 2.2 million new cases reported globally, has high morbidity and mortality rates [60,61]. Small-cell lung cancer (SCLC) is characterized by low differentiation, rapid growth, and extensive metastasis, making it one of the tumors with both a high malignant degree and mortality [62]. Due to these reasons, there is a constant need to develop better treatment plans for patients battling SCLC. Recently, a clinical study showed that patients with advanced SCLC respond well when treated with CPA plus vinorelbine [23]. Moreover, quality of life was improved, with decreased levels of inflammation.

The use of CPA has expanded beyond hematological and solid malignancies and has emerged as a treatment for autoimmune disorders such as rheumatoid arthritis [63]. Post-transplant CPA administration, developed as a prophylactic measure in graft-vs.-host disease (GVHD) management, has displayed potential superiority over standard prophylactic regimens [24]. Treatment with a low dose of CPA has demonstrated additional immune modifying properties through its impact on regulatory T cells (Tregs), which are a specialized population of T cells acting to suppress the immune response, thereby important in maintaining homeostasis and self-tolerance [64]. Infiltration of tumor cells with Tregs has been increasingly associated with impaired prognosis in various cancers, making it imperative to evaluate therapeutic strategies to address this concern [65]. A phase I clinical study involving metastatic renal cell carcinoma patients under a metronomic dose of CPA in combination with everolimus reported a transient decline in Treg numbers within 2 weeks of treatment [66]. This temporary depletion of Tregs by CPA can result in an elevation of tumor-reactive T cells, which boost the anti-tumor immune response. Although the mechanism underlying the effect of CPA on Tregs has yet to be clearly defined, potential reasons include the lack of efflux transporter expression in Tregs, which prevents them from pumping intracellular CPA out.

Collectively, CPA has been extensively used to treat various cancers, as well as severe autoimmune and inflammatory disorders. Upon bioactivation, this alkylating agent disrupts DNA replication in rapidly dividing cells. Impressively, more than 60 years since its discovery, CPA remains a prevalent and essential drug in clinical practice today.

#### 2.1.2. Mechanism of Bioactivation

**Figure 2:**
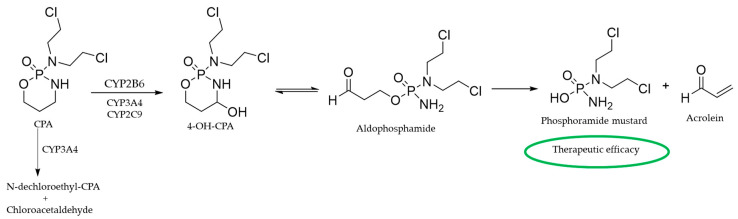
Schematic representation of metabolic bioactivation of CPA. CPA undergoes CYP2B6 catalyzed hydroxylation reaction to generate 4-OH-CPA. 4-OH-CPA rapidly undergoes spontaneous β-elimination, forming the biologically active phosphoramide mustard and the by-product acrolein. CYP3A4 can also mediate the conversion of CPA to N-dechloroethyl-CPA and chloroacetaldehyde. CPA: cyclophosphamide, 4-OH-CPA: 4-hydroxy CPA.

The chemical design of CPA was based on a rationale that tumor cells highly express phosphoamidase, an enzyme that is responsible for the hydrolysis of the phosphorous–nitrogen bond, enabling the release of the cytotoxic nitrogen mustard [5]. However, it was observed that CPA was effective only in vivo and failed to exhibit cytotoxicity in vitro, suggesting that the principal mode of CPA bioactivation might not be phosphoamidase dependent. It was later illustrated that CPA bioactivation is mediated by microsomal enzymes in the presence of cofactors such as nicotinamide adenine dinucleotide phosphate (NADPH). As indicated in Figure 2, 4-hydroxy-CPA (4-OH-CPA) is the initial product of CPA metabolism, which then undergoes ring opening to produce aldophosphamide [67]. A spontaneous β elimination leads to the decomposition of aldophosphamide to generate phosphoramide mustard, the single biologically active alkylating metabolite, and acrolein. An alternative metabolic pathway of CPA consists of a dechloroethylation reaction that generates products that lack any alkylating feature [68]. Clarke et al. utilized rat liver microsomes, reconstituted P-450 enzyme systems, and antibody inhibition experiments to identify the P-450 form PB-4 (CYP2B1, the rat analog of human CYP2B6) as the major hepatic monooxygenase catalyst mediating CPA 4-hydroxylation reaction in phenobarbital-induced rat liver microsomes [69].

In the late 1990s, Roy et al. determined the contribution of specific P450 towards the activation of CPA, utilizing a substrate-activity method based on liver microsomal P450 profile and the cDNA-expressed enzyme activities [70]. This study identifies that CYP2B6 catalyzes 48–57% of hepatic microsomal CPA 4-hydroxylation, which was further confirmed using a highly specific CYP2B6 monoclonal antibody. Furthermore, CYP2B6 exhibits a high K_m_/V_max_ ratio, suggesting its strong kinetic preference for CPA activation, making it the primary enzyme catalyzing the rate-limiting CPA hydroxylation reaction [70]. While CYP2B6-mediated CPA hydroxylation is the key reaction facilitating the formation of the DNA-alkylating mustard, CPA can also undergo CYP3A4-mediated side-chain oxidation to form the inactive metabolites N-dechloroethyl-CPA and chloroacetaldehyde [71]. In summary, metabolic bioactivation of CPA begins with the rate-limiting hydroxylation reaction to form 4-OH-CPA; this metabolite exists in equilibrium with its tautomer, aldophosphamide. CYP2B6 primarily catalyzes the conversion of CPA to 4-OH-CPA, with relatively minor contributions from CYP3A4, CYP2C9, and CYP2C18. The spontaneous β-elimination reaction of aldophosphamide yields the biologically active metabolite phosphoramide mustard, along with the release of the by-product acrolein.

#### 2.1.3. Off-Target Toxicity

Cardiac tissue injury has been known to occur with the use of alkylating agents. The incidence of CPA-related cardiotoxicity varies between 7 and 28% [27]. CPA-induced cardiotoxicity is dose dependent, where a dose greater than 150 mg/kg usually results in cardiac damage. Cardiac decompensation and cardiac myopathy occur in patients receiving a dose > 170–180 mg/kg for 4–7 days [72]. This dose can lead to further complications such as congestive heart failure, arrhythmias, and cardiac tamponade. CPA is known to cause intrapapillary microemboli, which can lead to ischemic damage. Ultrastructural examination of the tissue also showed multifocal myocardial necrosis, the presence of fibrin strands in the interstitium, disrupted and aggregated mitochondrial cristae, and degenerated myocardial tissue [27]. CPA treatment reduces antioxidant levels, which triggers oxidative stress in cardiomyocytes, contributing to its cardiac side effects. CPA-mediated mitochondrial injury diminishes ATP production, further damaging myocardial cells [27]. Another common side effect of CPA is hemorrhagic cystitis and bladder inflammation, which is primarily due to the formation of acrolein. Mesna is quite often prescribed to patients treated with CPA as a prophylactic. Interestingly, low-dose CPA has also been found to be associated with concerns such as hyponatremia, which could be directly mediated through a tubular effect [29].

CPA use is also associated with nausea, vomiting, and diarrhea, affecting the treatment tolerance and prognosis of patients [28]. Intestinal damage can occur by downregulating or altering the distribution pattern of tight junction proteins such as occludin, leading to enlarged intercellular spaces, compromising intestinal integrity [73]. The phosphoramide mustard formed from CPA is known to target rapidly proliferating cells, which makes the intestinal epithelial cells vulnerable to the effects of CPA. Studies have shown that CPA-related gastrointestinal toxicity is site selective, whereby damage primarily occurs in the small intestine but not in the large intestine [74]. This observation further implies that CPA-induced small-intestinal toxicity is related to regional CPA metabolism that is closely associated with intestinal CYP expression. Substantial variation in the expression of drug-metabolizing enzymes has been observed in different gastrointestinal tract segments [75,76]. Given the widespread intestinal toxicity caused by CPA, it would be clinically important to explore the contribution of intestinal CYPs toward CPA biotransformation.

CPA can also cause hepatotoxicity, which manifests as an elevation of alanine aminotransferase and aspartate transferase. Liver damage typically occurs at the higher CPA dose used for bone marrow transplantation (2–4 gm/m^2^), while the CPA dose given for immunosuppressive purposes does not exhibit significant hepatotoxicity [77]. Recently, Zhang et al. demonstrated that mice treated with CPA for five consecutive days demonstrated a remarkable increase in the levels of superoxide dismutase in the liver [78]. This antioxidant enzyme is involved with scavenging oxygen free radicals. Their enhanced formation, along with a rise in malondialdehyde concentration, is indicative of oxidative damage leading to lipid peroxidation.

The non-selective cytotoxicity of CPA enables it to interfere with the proliferation and differentiation of macrophages and lymphocytes, resulting in the killing of immune cells and thereby weakening the immune system [30]. Administration of CPA has been shown to reduce the secretion of Th1 and Th2 cytokines, leading to a change in Th1/Th2 bias in mice [79]. The use of CPA has also led to a decrease in spleen and thymus indices, which are significant markers of the immune state of an organism [80]. A decline in immunocompetence can be a major concern with CPA treatment, and bone marrow function, including leukocyte count, should be carefully evaluated in patients subjected to long-term CPA therapy. Thus, these various toxicity concerns associated with CPA make it imperative to monitor patient condition during treatment to ensure that the dosage can be appropriately adjusted if the patient experiences severe toxic effects.

### 2.2. Dacarbazine

Dacarbazine is an alkylating agent and a cell cycle non-specific synthetic analog of the naturally occurring purine precursor 5-amino-1H-imidazole-4-carboxamide [81]. Dacarbazine, also known as imidazole carboxamide, was approved for cancer treatment in 1975. Mechanistically, dacarbazine inhibits purine metabolism and nucleic acid synthesis and can hence hinder DNA and RNA synthesis, suppressing cell growth. This alkylating drug has been used to treat cancers, including melanoma, Hodgkins’s lymphoma, Kaposi sarcoma, and soft-tissue sarcoma [82,83]. Intravenous administration of dacarbazine is preferred because its absorption, when given orally, is relatively slow.

#### 2.2.1. Clinical Significance

Different regimens of dacarbazine have resulted in remission in patients with glucagonoma syndrome, a rare condition causing excess glucagon secretion due to the formation of a neuroendocrine tumor (NET) in the pancreas [84]. Remission has been achieved clinically, evidenced by the regression of tumor metastases, as well as a decline in plasma glucagon levels. NETs are neoplasms that develop in specialized neuroendocrine cells. The occurrence of NETs has steadily increased globally from 4.90 per 100,000 persons in 2000 to 8.19 per 100,000 persons in 2018 [85]. NETs may grow slowly or aggressively and most frequently occur in the gastrointestinal tract (>60%), where the midgut region is comparatively more sensitive than the foregut and the hindgut [86]. Treatment depends on the type of tumor but quite often includes surgery, radiation, and chemotherapy. Metastatic pancreatic NETs typically have poor outcomes, and the primary treatment modality consists of mTOR inhibitors and antiangiogenic multi-kinase inhibitors. Both these treatment methods have helped in prolonging progression-free survival in patients with pancreatic NETs [49,87]. Investigators have previously assessed the potential of combining dacarbazine with other agents, including 5-fluorouracil and epirubicin, in NET patients without chemotherapy [31]. Patients with Merkel cell carcinoma and pancreatic NETs included in this study showed a complete response, while partial response was observed in patients with medullary thyroid carcinoma and gastroenteropancreatic and carcinoid tumors. To evaluate the efficacy of low-dose dacarbazine administration, a retrospective study analyzing 75 patients with NETs of pancreatic origin was conducted between 1998 and 2013 [88]. In this study, 650 mg/m^2^ of dacarbazine was administered intravenously over 60 min every 4 weeks. The objective response rate was 32% for patients with pancreatic NETs, 39% of patients documented stable disease, and the median progression-free survival was 7 months. Moreover, the treatment was well tolerated, with only one case of grade 3 toxicity (vomiting) being reported among the patients receiving dacarbazine [88]. A more recent prospective single-arm trial investigated the therapeutic effect of dacarbazine in combination with ramucirumab, a vascular endothelial growth factor (VEGF) receptor-2 inhibitor, in patients with progressive well-differentiated metastatic pancreatic NETs [89]. This trial is expected to provide crucial data evaluating the new drug combination as a potential treatment strategy for pancreatic NET patients who are resistant to frontline therapy.

Dacarbazine has been the first-line treatment employed in clinical settings to manage malignant melanoma, a severe form of skin cancer originating in skin cells called melanocytes found in the upper layer of the skin [90]. This advanced and aggressive form of metastatic melanoma constitutes 4% of all dermatological cancers, with a high mortality rate [91]. Response rates with dacarbazine when used as a single agent are disappointing, ranging between 10 and 20%, with a complete response only seen in fewer than 5% of patients [92,93]. To help provide clinically significant improvements in the survival of patients battling metastatic melanoma, targeted therapy such as monoclonal antibodies, BCL-2 antisense oligonucleotide drugs, and kinase inhibitors are often included in the treatment approach. A meta-analysis of nine randomized control trials was conducted to compare the overall response between dacarbazine alone and dacarbazine-based treatment plans. This extensive study involving 2481 patients exhibited that dacarbazine-based combination therapies modestly improved the overall response and the 1-year survival rate [34]. One of the primary reasons for poor long-term survival of metastatic melanoma patients can be attributed to resistance towards drug-induced apoptosis [94,95]. The addition of a Bcl-2 inhibitor, oblimersen, to dacarbazine improved the 24-month progression-free survival [32]. Thus, this study indicates that the problem of chemoresistance could be mitigated by markedly reducing the Bcl-2-dependent anti-apoptotic effect. In a randomized phase II double-blind trial consisting of advanced melanoma patients, dacarbazine in conjunction with a Raf kinase inhibitor resulted in an increase in median time to progression and progression-free survival compared to the control group [96].

Hodgkin lymphoma is a type of cancer affecting the lymphatic system and develops when lymphocytes mutate and multiply uncontrollably. The standard approach to the treatment of Hodgkin lymphoma consists of ABVD (doxorubicin, bleomycin, vinblastine, and dacarbazine) followed by 20 Gy involved-field radiotherapy (IFRT) [97]. A randomized multi-center trial (HD13) was conducted to determine if dacarbazine can be omitted from the treatment regimen without compromising therapeutic efficacy [33]. This extensive study clearly illustrated that patients who were administered the regimen lacking dacarbazine had a greater number of relapses. Recently, it has been illustrated that replacing procarbazine with dacarbazine to treat Hodgkin lymphoma can help reduce hematopoietic stem cell and gonadal toxicity without any significant loss of clinical efficacy [98]. Another randomized trial assessing the effect of dacarbazine-based treatment on early-stage unfavorable classical Hodgkin lymphoma demonstrated favorable 1- and 3-year clinical outcomes among patients, with no major adverse effects [99]. The use of dacarbazine in another phase II study for classical Hodgkin lymphoma exhibited encouraging clinical efficacy, with a complete response rate of 88% [100]. Growing evidence from various clinical studies has clearly established this alkylating agent as a critical component of multiple regimens for Hodgkin lymphoma treatment.

#### 2.2.2. Mechanism of Bioactivation

**Figure 3:**
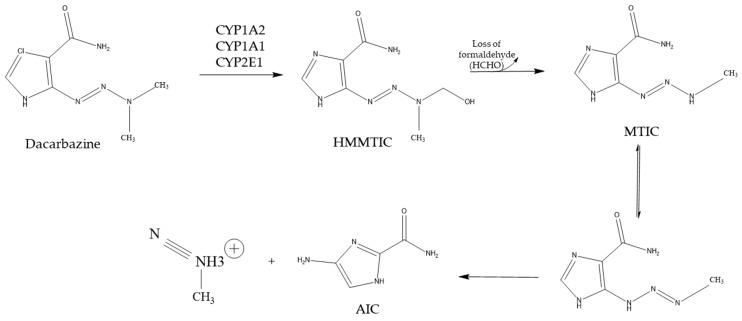
Schematic illustration of metabolic bioactivation of dacarbazine. Dacarbazine undergoes oxidation catalyzed primarily by CYP1A2 to generate the carbinolamine HMMTIC. MTIC formation occurs after loss of formaldehyde. Elimination of the methyl group from MTIC leads to the formation of AIC. HMMTIC: 5-[3-hydroxy methyl-3-methyl-triazen-l-yl]-imidazole-4-carboxamide, MTIC: 5-[3-methyl-triazen-1-yl]-imidazole-4-carboxamide, AIC: aminoimidazole carboxamide.

Dacarbazine is a triazene-based prodrug that needs to be bioactivated before exhibiting therapeutic effects. As shown in Figure 3, dacarbazine first undergoes a CYP-mediated N-demethylation to form the reactive species 5-[3-hydroxy methyl-3-methyl-triazen-l-yl]-imidazole-4-carboxamide (HMMTIC). There is a loss of formaldehyde from this initial product, which yields 5-[3-methyl-triazen-1-yl]-imidazole-4-carboxamide (MTIC). MTIC undergoes rapid decomposition to generate the major plasma and urinary metabolite aminoimidazole carboxamide (AIC) and the reactive species methane diazohydroxide, which produces molecular nitrogen and a methyl cation that is responsible for methylating guanine at the O-6 position, resulting in major cytotoxicity [101,102].

Reid et al. utilized chemical inhibition to characterize the specific CYP enzymes involved with the bioactivation of dacarbazine [103]. In this study, α-naphthoflavone, quercetin, phenacetin, chlorzoxazone, and disulfiram potently inhibited AIC formation, indicating the contribution of CYP1A, CYP2E1, and CYP3A enzymes towards dacarbazine metabolism. Interestingly, inhibition by α-naphthoflavone was essentially complete (>85%) at much lower concentrations, around 0.2 µM, insinuating CYP1A2 as the predominant enzyme involved in dacarbazine bioactivation [103]. Moreover, >75% dacarbazine metabolism was inhibited in microsomes incubated with human CYP1A2 antibody, corroborating the predominant role of CYP1A2 [103]. The correlation coefficient for the relationship between AIC formation and CYP1A2 index substrate reactions such as N^3^-demethylation and 7-ethoxyresorufin O-dealkylation was the highest (r > 0.94) [103]. The role of CYP1A2 was also underscored when α-naphthoflavone abrogated the correlation between CYP1A2-positive control reaction and dacarbazine metabolism. To further verify and confirm the individual isoenzymes involved in the bioactivation of dacarbazine, cDNA-expressed recombinant human CYPs were utilized. While all CYP1A1, CYP1A2, and CYP2E1 enzymes catalyzed dacarbazine biotransformation, CYP1A1 activity (3.62 ± 0.75 pmol AIC formed/pmol P450/min) is 3-fold greater than that of CYP1A2 (1.26 ± 0.24 pmol AIC formed/pmol P450/min) and 16-fold higher than that of CYP2E1 (0.22 ± 0.04 pmol AIC formed/pmol P450/min). Saturable enzyme kinetics for CYP1A1 and CYP1A2 were not observed in human microsomal systems, so individual cDNA enzymes were used to define the enzyme kinetics of dacarbazine N-demethylation. The k_m_ and V_max_ values for CYP1A1 were 595 ± 111 µM and 10.2 ± 2.1 pmol product/pmol P450/min, respectively. The k_m_ and V_max_ values for CYP1A2 were 659.6 ± 88 µM and 13.8 ± 2.4 pmol product/pmol P450/min, respectively. The kinetic data analysis for CYP2E1 revealed a K_m_ greater than 2.8 mM, indicating a low affinity of CYP2E1 for dacarbazine metabolism, which might play a secondary role compared to CYP1A. In conclusion, CYP1As are the principal enzymes that catalyze the bioactivation of dacarbazine in the liver. At the same time, CYP2E1 may participate under conditions where CYP1A expression might be low or in cases where dacarbazine concentration might saturate CYP1A.

#### 2.2.3. Off-Target Toxicity

Dacarbazine can cause gastrointestinal toxicity, including nausea, vomiting, and the sudden onset of abdominal pain. Bone marrow suppression effects such as leukopenia and thrombocytopenia are also reported with dacarbazine use. Cardiac-related effects, such as a fall in blood pressure, can also occur in patients treated with dacarbazine [35]. Additionally, after a second or third round of dacarbazine-based chemotherapy, distinctive clinical and pathological changes occur in the liver, indicating severe acute sinusoidal obstruction syndrome [36]. A dose-dependent increase in the necrosis of hepatocytes was observed with dacarbazine. One possible mechanism that leads to hepatotoxicity could be the potential induction of microsomal enzymes by dacarbazine, which leads to the generation of toxic metabolites [37]. Consequently, liver damage caused by dacarbazine can also be reflected in rapidly rising levels of serum aminotransferases. Centrilobular necrosis with occlusion of sinusoids and small and large hepatic veins has been observed following liver biopsy and necropsy. Local ischemia can also cause hepatic infarcts in some instances. Dacarbazine may also result in eosinophilic infiltrations and peripheral eosinophilia. Overall, chemotherapeutic doses of dacarbazine can cause various off-target effects that highlight the importance of careful monitoring and management during clinical use of this alkylating drug.

### 2.3. Duocarmycin

Duocarmycins are natural products isolated from Streptomyces and known for their extreme cytotoxicity. Duocarmycins belong to the cyclopropylpyrroloindole class of drugs and are highly potent, with IC_50_ ranging in the picomolar to nanomolar ranges [104,105]. Being extremely potent under systemic exposure, the scaffold of duocarmycins has been re-engineered to develop prodrugs that can be bioactivated to generate a cytotoxic metabolite. Duocarmycin analogs have also been utilized as payloads to develop ADCs. After binding the minor groove of DNA at the AT-rich region and alkylating adenine residue at the N3 position, duocarmycins cause DNA strand break and subsequent cell death by apoptosis, impacting both proliferating and non-proliferating cells [105,106].

#### 2.3.1. Clinical Significance

The first duocarmycin analog, CC-1065, was reported in 1978 after being isolated from Streptomyces zelensis. CC-1065 was effective against leukemia and melanoma cell lines in vitro by inhibiting DNA synthesis and cell cycle progression [107]. However, toxicological studies revealed that the dose of CC1065 required to exert antitumor effects can cause delayed severe toxicity and death in experimental animals [108]. Due to this, several different analogs of CC-1065 were developed where the structural features, such as DNA-binding moiety and the spirocyclic cyclopropylpyrroloindole moiety, were maintained. One such analog is ICT2700, whose cytotoxic effect is mediated by CYP1A1 metabolic bioactivation [109]. The cytotoxic effect of ICT2700 was investigated in CHO cells stably transfected with CYP1A1 in which ICT2700 demonstrated cytotoxicity at picomolar concentrations. In contrast, the anticancer effect was markedly weaker in A2780 ovarian cancer cells (IC_50_ > 1000 nM), which lack CYP1A1 expression [109].

There is a high similarity in the sequence identity for the substrate recognition sites between CYP1A1 and CYP2W1 [110]. This similarity led researchers to hypothesize that certain CYP1A1 substrates, specifically those containing cyclopropylpyrroloindole functional groups, could also be potential CYP2W1 substrates [39]. From a chemotherapeutic standpoint, this could be beneficial, since CYP2W1 is highly expressed in approximately 30% of primary colorectal cancers (CRC) but exhibits minimal expression in normal tissue [39]. Travica et al. utilized CYP2W1-transfected cell lines and a human colon cancer murine xenograft model to study the efficacy of ICT2700 derivatives such as ICT2705, ICT2706, and ICT2726 [38]. In SW480-2W1 (CYP2W1 stable line) and Colo320-2W1 (CYP2W1 transient expression) cells, ICT2705 and ICT2706 treatment led to a concentration-dependent decrease of cell viability in both the cell lines, while ICT2726 failed to induce cytotoxicity. The efficacy of ICT2706 was further confirmed in a SW480-2WI xenograft model established in SCID mice [38]. These findings are encouraging and can be beneficial towards the gene-directed enzyme prodrug therapy (GDEPT) strategy, improving efficacy and mitigating toxicity concerns in CRC patients. Recent in vitro studies indicated that duocarmycin can also induce cell cycle arrest and proliferation inhibition in acute myeloid leukemia cells [111].

#### 2.3.2. Mechanism of Bioactivation

**Figure 4:**
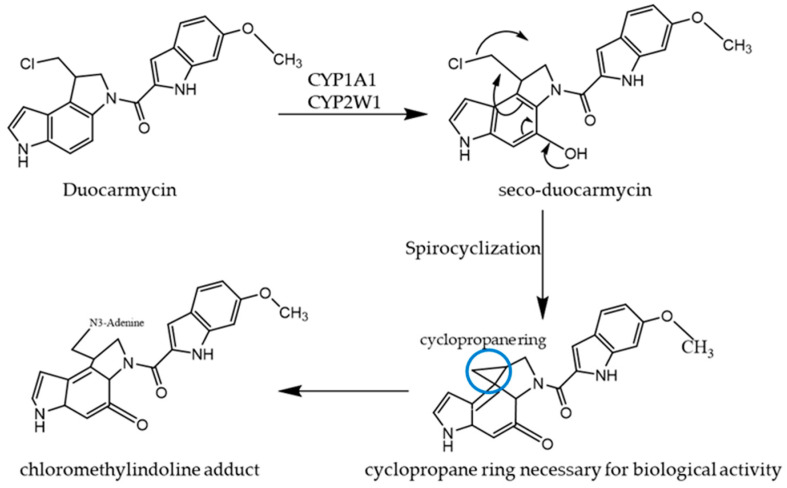
Schematic representation of metabolic bioactivation of duocarmycin. Duocarmycin hydroxylation can be mediated by CYP1A1 and CYP2W1. Seco-duocarmycin undergoes spontaneous spirocyclization to form a cyclopropane ring, which can alkylate N^3^ residue of adenine in DNA.

Duocarmycins are a class of potent anticancer antibiotics whose scaffold can be re-engineered to design prodrugs that require CYP1A1- and CYP2W1-mediated bioactivation to exert their pharmacological action. High expression of CYP1A1 in lung and bladder tissues allows duocarmycins to be utilized for lung and bladder cancer [112,113]. As demonstrated in Figure 4, the first step in the bioactivation of duocarmycin is its CYP-mediated hydroxylation to form *seco*-duocarmycin. The deep-embedded chloromethylindoline fragment in the hydroxylated *seco*-duocarmycin undergoes spontaneous spirocyclization. This reaction, which transforms *seco*-duocarmycin to active duocarmycin, is referred to as Weinstein spirocyclization [114]. The step of spirocyclization helps release chlorine, which generates a constrained cyclopropane that is appropriately located to form a covalent adduct with N^3^-adenine upon binding the indole-containing DNA-binding subunit. In the case of ICT2700, the phenolic oxygen is absent, preventing the intramolecular formation of cyclopropane, critical for alkylating DNA. Thus, CYP-dependent site-specific hydroxylation is crucial for the bioactivation of duocarmycin-based precursors [38].

Studies from several laboratories have provided strong evidence indicating that duocarmycin and its analogs afford the highest rate of reactivity upon binding to the deep minor groove of AT-rich DNA sequences. Additionally, it has been established that DNA minor groove binding agents are preferred in AT base pairs because they favor hydrophobic interactions. N^3^ of an adenine residue is responsible for the nucleophilic attacks at the least substituted position of the spirocyclopropyl ring of duocarmycin, leading to DNA alkylation [115]. The adduct formation between duocarmycin and adenine residue is of central importance in triggering duocarmycin’s antitumor effect, primarily due to the stability of the formed adduct and the inability of the standard DNA repair machinery to correct these damages [115]. The X-ray crystal structure of duocarmycin and some of its analogs in ground state conformation have displayed two subunits with three torsion angles in the linker. The two subunits twist with respect to each other upon binding to the minor groove to maximize hydrophobic interaction with DNA [115]. The subsequent loss of the vinylogous amide stabilization of the cyclopropyl group, which occurs by twisting the linker between the two subunits, also facilitates the activation of the ligand. Such binding-induced and shape-dependent catalysis constitutes an essential feature of duocarmycin’s in situ mechanism of action, which is not commonly exhibited by other DNA-binding agents. However, it offers considerable potential to design functional analogs.

#### 2.3.3. Off-Target Toxicity

Due to their extremely potent cytotoxicity, to this end, there are no FDA-approved duocarmycin analogs for clinical use. The major side effects reported with duocarmycin use include hepatotoxicity and myelosuppression. SYD985, a second-generation ADC-containing duocarmycin, can cause fatigue and conjunctivitis in patients [40]. MGC018, an investigational ADC with a duocarmycin payload linker, has shown hyperpigmentation and erythema [41]. A considerable decrease in peripheral leukocytes and erythrocytes was also observed. Liver function parameters, including alanine aminotransferase, aspartate aminotransferase and alkaline phosphatase, were elevated with the use of MGC018 [41]. Clinical trials conducted with MGC018 have recently raised safety concerns regarding this agent [42]. The cleavable nature of duocarmycins could make them a promising target for cleavable linkers for the development of anticancer ADCs, which can drastically reduce toxicities in nonmalignant cells.

### 2.4. Other Alkylating Prodrugs

**Figure 5:**
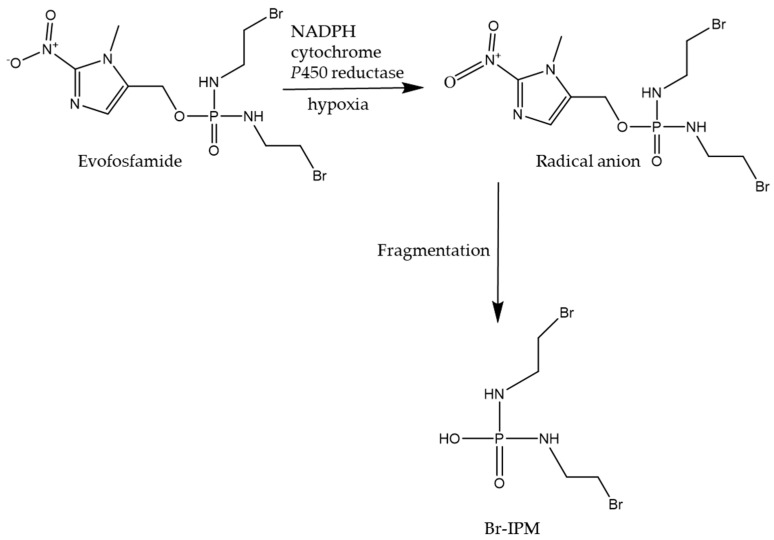
Schematic representation of metabolic bioactivation of evofosfamide. The nitroimidazole prodrug when exposed to hypoxic conditions releases a radical anion, which then undergoes fragmentation to form Br-IPM. Br-IPM: bromo-isophosphoramide mustard.

Alkylating agents continue to be a cornerstone in chemotherapy due largely to their ability to covalently transfer alkyl groups to nucleophilic moieties in DNA under physiological conditions. Apart from the abovementioned alkylating drugs, there is a battery of alkylating agents that are employed for both hematological and solid malignancies [116]. Some chemotherapeutic prodrugs are activated by hypoxia in the presence of reducing agents or non-CYP P450s. One such prodrug is evofosfamide, a nitroimidazole prodrug of bromo-isophosphoramide mustard (Br-IPM). This second-generation hypoxia-activated prodrug can diffuse into hypoxic regions in the absence of activation by DT diaphorase [117]. As shown in Figure 5, upon exposure to hypoxic conditions, evofosfamide forms a radical anion that undergoes fragmentation to release the alkylating agent Br-IPM in the presence of intracellular reductases. This mode of activation would be beneficial to target cancers such as glioblastomas because of their hypoxic nature. A single-arm phase II study designed to explore the safety and efficacy of evofosfamide and bevacizumab in glioblastoma patients refractory to bevacizumab reported a marked improvement in progression-free survival compared to bevacizumab treatment [43]. Investigators have also developed a prodrug by combining two FDA-approved drugs, namely chlorambucil and cisplatin [118]. Improving cisplatin solubility has been shown to reduce its resistance by improving passive diffusion through the cell membrane [118]. A prodrug containing oxidized cisplatin and chlorambucil can offer a promising strategy to circumvent lipophilicity issues and improve therapeutic efficacy, as both are antineoplastic agents attacking DNA. After entering the cells, chlorambucil and cisplatin will be released from the prodrug formula with the help of reducing agents, such as ascorbic acid and glutathione. When this prodrug was assessed in an in vivo xenograft model using MDA-MB-231 tumor-bearing mice, tumor growth was significantly inhibited [119]. Moreover, compared to cisplatin, which is highly protein-bound and can accumulate in tissues such as the kidneys, this study showed that the prodrug was well tolerated and did not result in any appreciable tissue damage. Over the years, liposomes have become an attractive vehicle for drug delivery since they display enhanced permeability and retention effects and their ability to reduce both acute and delayed side effects of anticancer agents. Promitil^®^, a PEGylated liposomal prodrug of mitomycin c, can be activated by thiolytic cleavage to release this potent alkylating agent. A phase I study of Promitil^®^ showed that durable stabilization was attained, with a subsequent decline in the level of carcinoembryonic antigen, a tumor marker, in colon cancer patients [120]. Developing these different prodrugs can help achieve improved selectivity, thereby mitigating some of the limitations that encumber conventional chemotherapy.

## 3. Non-Alkylating Prodrugs

Non-alkylating drugs can function either as antimetabolites, antitumor antibiotics, or plant alkaloids. Certain non-alkylating drugs influence the maintenance of DNA structure. Topoisomerase 1 (Top1), a primary target of irinotecan, is a crucial enzyme that helps to remove torsional stress [121]. Additionally, this class of chemotherapeutic agents can regulate genetic and epigenetic processes such as histone modification. In this section, we will summarize the clinical significance, activation mechanisms, and off-target toxicity of 5-Fluorouracil, irinotecan, romidepsin, and, to a lesser extent, PRO962 (Table 2).

### 3.1. 5-Fluorouracil

5-Fluorouracil belongs to a group of chemotherapeutic drugs referred to as anti-metabolites and can exert its cytotoxic effect following activation by a series of biochemical intracellular pathways. As a pyrimidine analog, misincorporating 5-Fluorouracil into RNA and DNA negatively impacts cell proliferation and eventually leads to cell death [122]. 5-Fluorouracil is also known to inhibit thymidylate synthase (TS), resulting in deoxynucleotide (dNTP) pool imbalance and increased levels of deoxyuridine triphosphate (dUTP), which ultimately causes DNA damage.

#### 3.1.1. Clinical Significance

The fluorinated pyrimidine, 5-Fluorouracil, was first synthesized by Charles Heidelberger in 1957 [9]. 5-Fluorouracil inhibits TS, an enzyme critical for the de novo synthesis of thymidylate, a necessary component for DNA replication and repair. Inhibition of TS blocks deoxythymidine synthesis, which subsequently leads to depletion of deoxythymidine triphosphate (dTTP), leading to deoxynucleotide pool imbalance, ultimately disrupting DNA synthesis and repair [123].

**Table 2:** Non-alkylating prodrugs and their cancer targets, bioactivation mechanisms, and side toxicities.

Non-Alkylating Prodrug	Target Cancer	Bioactivation Mechanism	Off Target Toxicity
5-Fluorouracil	Adenocarcinomas and head and neck cancer [124,125,126]	Orotate phosphoribosyltransferase, uridine kinase, uridine phosphorylase, thymidine phosphorylase, and thymidine kinase	Angina, myocardial infarction, heart failure, encephalopathy, seizures, coma, and peripheral nerve damage [127,128,129]
Irinotecan	Metastatic colorectal cancers, ovarian cancer, and small-cell lung cancer [130,131,132,133]	Carboxylesterases	Diarrhea, lacrimation, salivation, visual disturbances, and neutropenia [134,135,136]
Romidepsin	Cutaneous T-cell lymphoma, peripheral T-cell lymphoma, and prostate cancer [137,138,139,140]	Disulfide bond reduction	ST/T wave flattening, ST depression, ST inversion, QTc prolongation, and electrolyte imbalance [141,142,143]
PRO962	Epidermoid carcinoma	pH < 7.0	Skin changes, weakness, and diarrhea [144,145]

In the 1960s, the FDA approved the systemic use of 5-Fluorouracil for treating gastric adenocarcinoma, pancreatic adenocarcinoma, breast adenocarcinoma, and colorectal adenocarcinoma. Studies conducted in human-derived pancreatic adenocarcinoma cell lines such as PANC-1, HPAF-II, and BxPC-3 revealed that lurbinectedin, an antineoplastic agent, enhanced the synergistic cytotoxic effect exerted by irinotecan and 5-Fluorouracil [124]. The enhanced cytotoxicity demonstrated by the triple combination of drugs was attributed to DNA damage, replicative stress, and upregulation of cytokines related to apoptotic pathways [124]. Using pancreatic cell lines, Zhao et al. investigated the impact of 5-Fluorouracil on p38, which is known to play a crucial role in tumor development and stemness maintenance [146]. 5-Fluorouracil treatment caused cell cycle arrest at the S-phase in a dose-dependent manner. Additionally, this study demonstrated that tumor formation was suppressed and stemness declined, as indicated by a decrease in the positive expression of CD44 and CD133, which are markers of cancer stem cell maintenance.

Head and neck cancer (HNC) is the 7th most common type of cancer [147]. In total, 90% of HNCs are squamous cell carcinoma and heterogenous, which arise from the epithelial cells of the oral cavity, pharynx, and larynx [148]. The standard form of treatment for this heterogeneous type of cancer includes surgery, radiation, and chemotherapy. 5-Fluorouracil has also been investigated as part of a concomitant chemoradiotherapy strategy for the treatment of patients with locoregionally advanced HNC [125]. A randomized phase III study was conducted, including previously untreated patients with locoregionally advanced disease. [126]. This study revealed that patients receiving a combination of 5-Fluorouracil, cisplatin, and docetaxel demonstrated clinically significant improvement outcomes compared to patients treated only with cisplatin and 5-Fluorouracil. Patients treated with this triple medication combination had a 28% decrease in risk of disease progression or death and a median overall survival of 18.8 months in comparison to 14.5 months with cisplatin and 5-fluorouracil treatment. Another study in Japan suggested that administering 80 mg/m^2^ of cisplatin on day 1 and a 5-Fluorouracil dose of 800 mg/m^2^ per 24 h via continuous intravenous infusion is more effective for the palliative treatment of recurrent or metastatic squamous cell HNC [149]. A recent study evaluated the efficacy of administering a flat dose of 500 mg 5-Fluorouracil to treat HNC patients, using dose adjustments based on pharmacokinetic parameters instead of body surface area [150]. The results indicated that a positive response was obtained with a flat dose, and therapeutic dose monitoring based on the 5-Fluorouracil target area under the curve was effective in adjusting doses for under- and over-exposed patients.

#### 3.1.2. Mechanism of Bioactivation

**Figure 6:**
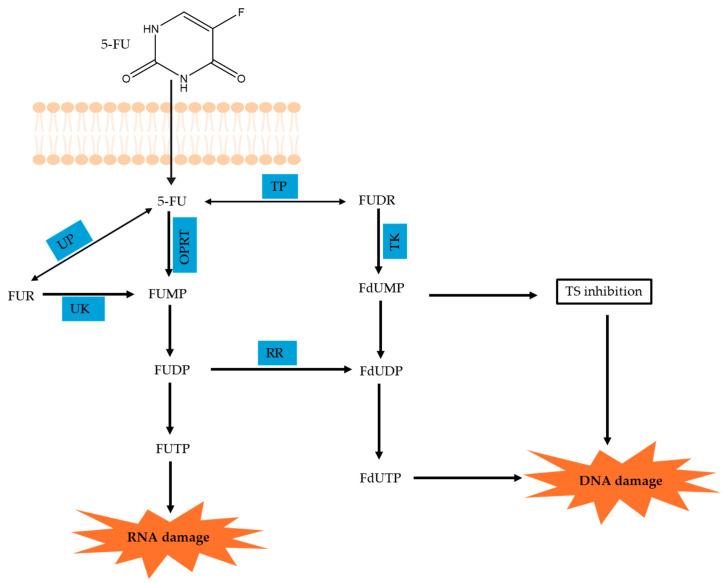
Schematic representation of bioactivation of 5-Fluorouracil. Following uptake, 5-FU undergoes multiple enzyme-catalyzed reactions to generate different active metabolites, which lead to TS inhibition that results in DNA damage. 5-FU: 5-Fluorouracil, FUDR: fluorodeoxyuridine, FUR: fluorouridine, FUMP: fluorouridine monophosphate, FUDP: fluorouridine diphosphate, FUTP: fluorouridine triphosphate, FdUMP: fluorodeoxyuridine monophosphate, FdUDP: fluorodeoxyuridine diphosphate, FdUTP: fluorodeoxyuridine triphosphate, TS: thymidylate synthase, TP: thymidine phosphorylase, UP: uridine phosphorylase, OPRT: orotate phosphoribosyltransferase, TK: thymidine kinase, UK: uridine kinase, RR: ribonucleotide reductase.

5-Fluorouracil is an analog of the nucleotide uracil with a substituted fluorine at the C-5 position. 5-Fluorouracil by itself does not exhibit any cytotoxicity, and its anticancer effects are exerted upon its interaction with phosphorylated sugars via enzymatically catalyzed reactions [122]. As illustrated in Figure 6, upon entering the cells via a facilitated transport system, 5-Fluorouracil is converted to various active metabolites such as fluorodeoxyuridine monophosphate (FdUMP), fluorodeoxyuridine triphosphate (FdUTP), and fluorouridine triphosphate (FUTP). These active metabolites can disrupt RNA synthesis and inhibit the action of TS [122]. Among others, the primary mechanism of 5-Fluorouracil activation lies in its conversion to fluorouridine monophosphate (FUMP). This can occur directly, with the reaction being catalyzed by orotate phosphoribosyltransferase (OPRT) using phosphoribosyl pyrophosphate (PRPP) as the cofactor [122]. Indirectly, 5-Fluorouracil can be activated by uridine phosphorylase (UP) to form fluorouridine (FUR), which is then converted to FUMP by uridine kinase (UK) [151]. FUMP undergoes phosphorylation to generate FUDP (fluorouridine diphosphate) and FUTP, with FUTP causing RNA damage. FUDP, on the other hand, can be converted to fluorodeoxyuridine diphosphate (FdUDP) by ribonucleotide reductase (RR), which can then form either FdUTP or FdUMP. Alternatively, 5-Fluorouracil can be converted to FdUMP via thymidine phosphorylase (TP) and thymidine kinase (TK). The resulting FdUMP can subsequently inhibit TS [122].

Dihydropyrimidine dehydrogenase (DPD) catalyzes the degradation of 5-Fluorouracil to dihydrofluorouracil. This reaction occurs predominantly in the liver, which leads to poor bioavailability and inconsistent pharmacokinetics of 5-Fluorouracil. This is one of the key reasons 5-Fluorouracil is conventionally delivered intravenously and a major factor influencing the design of 5-Fluorouracil prodrugs [152]. Tegafur is a 5-Fluorouracil prodrug that is classified as a DPD inhibitory fluoropyrimidne. Uracil is included in Tegafur in a 4:1 molar ratio because it helps improve its therapeutic index by enhancing the half-life of the released 5-Fluorouracil [153]. It has been reported that CYP2A6 is the principal enzyme responsible for releasing 5-Fluorouracil from Tegafur [154]. CYP2A6 mediates the hydroxylation at the 5′ position of the furan ring in Tegafur to form 5′-hydroxytegafur, which is spontaneously converted to 5-Fluorouracil. Interestingly, bioactivation of Tegafur to 5-Fluorouracil in human microsomes displayed a biphasic kinetic feature, with the K_m_ and V_max_ values for the high-affinity component at 0.43 ± 0.05 mM and 4.02 ± 1.70 nmol/mg/min, respectively. Additionally, the inclusion of coumarin, a CYP2A6-selective substrate, inhibited the formation of 5-Fluorouracil in human liver microsomes in a concentration-dependent manner [154]. The role of CYP2A6 was further confirmed, where the anti-CY2A6 antibody prevents the formation of 5-Fluorouracil by almost 90% in liver microsomes. However, it has also been shown that cytosolic 5-Fluorouracil can be formed by the activation of TP. Komatsu et al. demonstrated a K_m_ value of 1.8 ± 0.3 mM, which is 10-fold lower than the cytosolic value of 16 ± 4 mM for the formation of 5-Fluorouracil [155]. The study also investigated the influence of TP on the bioactivation of Tegafur, in that 5-Fluorouracil formation from Tegafur was significantly correlated with the activity of liver cytosolic TP [155]. The addition of tipiracil hydrochloride, a selective inhibitor of TP, efficiently prevented the cytosolic formation of 5-Fluorouracil [156]. Findings from this study suggest that the release of 5-Fluorouracil from Tegafur catalyzed by microsomal enzymes occurs at a low concentration, while the contribution of cytosolic TP mainly becomes evident at a higher Tegafur concentration.

#### 3.1.3. Off-Target Toxicity

5-Fluorouracil has been known to result in cardiovascular side effects, including angina, myocardial infarction, and heart failure. The administration of 5-Fluorouracil as an infusion can cause greater cardiotoxicity compared to a bolus regimen. A 24-h infusion of 5-Fluorouracil at 2600 mg/m^2^/week elevated the level of ammonia, leading to encephalopathy in cancer patients [127]. A potential explanation of this effect could be the accumulation of fluorocitrate, a by-product of 5-Fluorouracil metabolism, which can impact the Krebs cycle and further impair the ATP-dependent urea cycle [128]. 5-Fluorouracil can also induce neuropathy, leading to neurological adverse events such as cerebellar syndrome, ataxia, seizures, coma, and peripheral nerve damage. Additionally, 5-Fluorouracil inhibits the generation of new cells in the hippocampus, which is essential for brain plasticity and neural repair. Hence, a decline in cognitive function is associated with 5-Fluorouracil-mediated suppression of neurogenesis in the hippocampus [129]. Notably, DPD catalyzes the conversion of nearly 80% of the administered 5-Fluorouracil to its inactive metabolite in the liver, gut, and various other tissues. Oral administration of 5-Fluorouracil can result in erratic and unpredictable plasma concentrations of the drug and its metabolites, primarily due to the extensive variability in DPD activity. Severe fluoropyrimidine toxicity occurs in DPD deficiency, which leads to serious concerns of pancytopenia, desquamative skin lesions, acute kidney failure, and septic shock [157].

### 3.2. Irinotecan

Irinotecan, or CPT-11, is a water-soluble derivative of the plant alkaloid camptothecin. Irinotecan was first approved for use in Japan to treat cervical, lung, and ovarian cancers. Irinotecan interferes with the topological functions of cells that eventually affect DNA replication, transcription, chromosome segregation, and recombination [158].

#### 3.2.1. Clinical Significance

Irinotecan is a prodrug that primarily targets Top1, an essential nuclear enzyme involved in relaxing supercoiled DNA duplexes to help DNA replication and RNA transcription. The cleavable complex formed between DNA and the enzyme helps DNA to relax around the single-strand nick, which is later resealed by Top1. The active metabolite of irinotecan, SN-38, binds to this cleavable complex, stabilizing it, which leads to single-strand breaks that the enzyme cannot re-ligate [159]. It has been shown that cells in the S-phase are more sensitive to the cytotoxic effects of irinotecan than those in the G1-G2 phase [160]. This could imply that cytotoxicity is a consequence of the interaction between the cleavable complex and replication forks, which might be responsible for converting a single-strand break into a double-strand break [161].

Irinotecan is known to be a key component of both the first- and second-line treatment of metastatic CRC. The 5-year survival rate among these late-stage patients remains dismal. Both FOLFIRI and FOLFOXIRI regimens containing irinotecan have been shown to be equally efficacious in metastatic CRC treatment [130]. Recently, a meta-analysis study involving 4571 patients revealed that irinotecan- and oxaliplatin-based treatments could be employed as a first-line treatment strategy for metastatic CRC. Moreover, the combination of irinotecan with an anti-VEGF antibody resulted in improved progression-free survival [162]. CRC has been shown to have mutations in the MAPK pathway, including in BRAF. The commonly found V600E mutation in BRAF is associated with an aggressive cancer phenotype. Standard treatment options, including epidermal growth factor receptor (EGFR) inhibitors, are insufficient to treat patients with BRAF mutations. Various clinical studies utilizing concomitant administration of irinotecan, vemurafenib, and cetuximab have reported a 35% response rate with positive progression-free survival in treating BRAF^V600E^-mutated CRC [163,164]. Irinotecan plus 5-Fluorouracil/leucovorin can be beneficial to patients treated with palliative intent, enabling them to undergo surgical resection following chemotherapy [165]. Clearly, irinotecan plays a significant role in the treatment of this cancer type and can also be used as a neoadjuvant treatment strategy for patients undergoing surgical resection.

Irinotecan has also been used to treat ovarian cancer, which represents the 5th leading cause of cancer-related death among women [166]. The standard form of treatment consists of radiotherapy followed by platinum- and paclitaxel-based chemotherapy. In the case of patients that develop resistance to platinum-based chemotherapy, salvage chemotherapeutic regimens consisting of irinotecan and other drugs can help sustain treatment. A phase I/II study using irinotecan and gemcitabine showed a synergistic effect among taxane/platinum-resistant/refractory ovarian and primary peritoneal cancer patients, improving the overall response rate and the clinical benefit ratio. The clinical benefit ratio, a secondary endpoint of the study, takes into consideration complete and partial response, along with stable disease [167]. Another phase II study evaluating the efficacy of a tailored dose of irinotecan and gemcitabine achieved a disease control rate of 75% in 48% of patients with platinum-refractory/resistant ovarian or peritoneal cancers [131]. The progression-free survival and the overall survival rates from this study were 6.2 months and 16.8 months, respectively. The combination of irinotecan and lurbinectedin also revealed a potent synergistic effect in BRCA-mutated platinum-resistant ovarian cancer patients [132].

SCLC is a neuroendocrine carcinoma with distinct biological and clinical characteristics compared to other types of lung cancer. SCLC grows rapidly and frequently spreads to distant sites, making it aggressive. As most patients relapse within 1–2 years, resistance to standard treatment regimens remains a major challenge associated with the management of SCLC [168]. Hence, there is a constant need to evaluate different medications for second-line treatment of SCLC. A phase II study investigated irinotecan as a single agent for the treatment of SCLC patients experiencing relapse [133]. This study reported a disease control of 69% and achieved an overall response rate of 61% among patients experiencing relapse. Liposomal irinotecan, a liposomal-encapsulated formulation of irinotecan, is intended to improve pharmacokinetic and toxicological properties compared to conventional irinotecan [169]. A clinical study evaluated the efficacy, safety, and tolerability of liposomal irinotecan for SCLC. Phase II results showed encouraging anti-tumor activity of liposomal irinotecan [170]. Another trial compared liposomal irinotecan to topotecan in patients who relapsed after first-line platinum-based chemotherapy, reporting similar median overall survival for both treatments [171]. Previous studies have shown that liposomal irinotecan attains higher and sustained intratumoral levels of both the parent drug and its metabolite SN-38 relative to non-liposomal formulations [172,173]. Initial promising results with this innovative delivery strategy of irinotecan warrant further research that can support the use of liposome-based drug delivery of this topoisomerase inhibitor.

#### 3.2.2. Mechanism of Bioactivation

**Figure 7:**
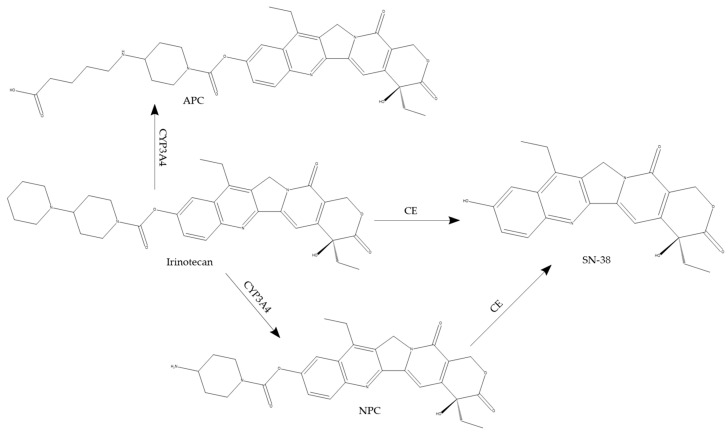
Schematic representation of bioactivation of irinotecan. CE mediates the hydrolysis of the bis-piperidine side chain of irinotecan to yield the active SN-38. CYP3A4 can also convert irinotecan to less active metabolites APC and NPC. CE can further NPC to form SN-38. APC: 7-ethyl-10-[4-N-(5-aminopentanoic acid)-1-piperidino] carbonyloxycamptothecin, NPC: 7-ethyl-10-(4-amino-1-piperidino) carbonyloxycamptothecin, CE: carboxylesterase.

Irinotecan is a pentacyclic alkaloid containing a bis-piperidine side chain that enhances its solubility. The lactone form of irinotecan is active, but it is impermeable through the lipid bilayer. Hence, it is inactive in its carboxylate form. As depicted in Figure 7, irinotecan undergoes cleavage of the ester bond at the C10 position mediated by carboxylesterase (CE) to release the pharmacologically active SN-38 [174,175]. Rivory et al. reported that deacylation could be the rate-limiting step in the hydrolysis of irinotecan by CE [174]. CEs are a group of serine esterases found in different animal tissues. CEs are known to metabolize various endogenous and xenobiotic compounds, including cholesterol ester, aspirin, and cocaine. CE1 and CE2 are the two primary forms of CEs involved in human drug metabolism [176]. CE1 is predominantly found in the liver, while CE2 is present in the small intestine, liver, colon, and kidney. Utilizing CEs purified from the human liver to analyze irinotecan hydrolysis resulted in a k_m_ of 42.7 ± 6.8 µM and 3.4 ± 1.4 µM for CE1 and CE2, respectively, and the V_max_ of CE2 was also 5-fold higher than that of CE1 [177]. This finding indicates that the rate of irinotecan conversion by CE2 is significantly greater than that by CE1 at pharmacologically relevant doses. The amount of SN-38 generated correlated with the cytotoxicity observed, with only 38% cell survival observed after incubation of irinotecan with CE2 in contrast to 88% cell survival following exposure to CE1. The critical role of CE2 in irinotecan bioactivation and the subsequent therapeutic effect of SN-38 generated was further established using a multiple myeloma-based xenograft mouse model [178]. Enhanced cytotoxicity of irinotecan towards multiple myeloma cells in vitro and in vivo was observed due to their overexpression of CE2. Data from these studies clearly depict that CE2 is the enzyme with higher affinity and higher velocity in comparison to CE1.

In vitro and molecular modeling studies confirmed that irinotecan could inhibit acetylcholinesterase activity [134]. Incubation of irinotecan with increasing concentrations of butyrylcholinesterase decreased SN-38 formation [179]. Metabolism of irinotecan to SN-38 was also inhibited in the presence of tacrine, a specific cholinesterase inhibitor [179]. It has been previously established that only 13% of the administered irinotecan dose is excreted unchanged in the urine, suggesting extensive metabolism of the drug [180]. In addition to SN-38 and SN-38 glucuronide (SN-38G), 7-ethyl-10-[4-N-(5-aminopentanoic acid)-1-piperidino] carbonyloxycamptothecin (APC) and 7-ethyl-10-(4-amino-1-piperidino) carbonyloxycamptothecin (NPC) are detected in the urine of patients treated with irinotecan [181]. Notably, formation of APC and NPC is mediated by CYP3A4 and CYP3A5, with CYP3A4 displaying a greater affinity [182]. Pharmacologically, APC and NPC are less active than SN-38, but NPC can be further hydrolyzed to SN-38 by CE. Due to the influence of CYP3A on irinotecan metabolism, its therapeutic efficacy can be altered in the presence of medications that modify the activity of this CYP enzyme.

#### 3.2.3. Off-Target Toxicity

The primary dose-limiting toxicity of irinotecan is diarrhea, although irinotecan-based treatment regimens have been generally categorized as moderate emetic risk chemotherapy. The cholinergic surge due to acetylcholinesterase inhibition is responsible for diarrhea that occurs within a few hours after irinotecan treatment [134]. Patients also experience lacrimation, salivation, and visual disturbances due to the impact of irinotecan on acetylcholinesterase. Late-onset diarrhea is unpredictable, with the underlying mechanism being SN-38-mediated apoptosis and crypt hypoplasia in the intestines, which lead to colon damage and mucin secretion [135]. Irinotecan-induced diarrhea can be life-threatening and is often treated using loperamide, a dopamine agonist. The phase II drug-metabolizing enzyme uridine diphosphate (UDP)-glucuronosyltransferase 1A1 (UGT1A1) plays a pivotal role in SN-38 glucuronidation and detoxification [183]. Patients with loss-of-function UGT1A1 polymorphisms are at greater risk of irinotecan toxicities. UGT1A1*28, a typical genotype of people with Gilbert’s syndrome, is characterized by a 30–50% decline in glucuronidation efficiency than the normal population [184]. In 2005, the FDA recommended that patients with UGT1A1*28 homozygous undergoing irinotecan treatment should receive a 30% lower dose than the standard dose [185]. This direct impact of UGT1A1 genetic variations on irinotecan has resulted in the growing relevance of pre-therapeutic testing for UGT1A1*28 and UGT1A1*6 in patients indicated for irinotecan treatment [186].

Neutropenia is another side effect associated with clinical use of irinotecan [136]. Neutropenia occurs when the neutrophil count in circulation falls below normal. Neutrophils are white blood cells that help the body fight infections; hence, irinotecan-induced neutropenia is a major concern as it increases the risk of infection among patients treated with this drug. Prolonged exposure to SN-38 in patients who are homozygous to UGT1A1*28 mutation has been known to result in this particular hematological toxicity [187]. Recently, a study conducted in Japan observed decreased incidences of severe neutropenia among patients who received a reduced dose of irinotecan [188]. As patients undergoing chemotherapeutic treatment already have a compromised immune system, these patients, when treated with irinotecan, need to be closely monitored to reduce the occurrence of severe infection.

### 3.3. Romidepsin

Romidepsin, or Istodax, is a cage-shaped bicyclic depsipeptide isolated from gram-negative bacteria. This selective histone deacetylase (HDAC) inhibitor, approved by the FDA in 2009, was used for treating cutaneous T-cell lymphoma (CTCL). The recommended dose is 14 mg/m^2^, administered intravenously over a 4 h period. Romidepsin decreases cyclin D1 and c-Myc expression while inducing p21. This induction triggers the inhibition of cyclin-dependent kinase (CDK) and dephosphorylation of the retinoblastoma protein (Rb), leading to cell cycle arrest [189].

#### 3.3.1. Clinical Significance

The reduced form of romidepsin is active, enabling the thiol side chain to coordinate with the zinc ion in the HDAC active site, thereby causing HDAC inhibition. HDACs play a role in removing acetyl groups from histone tails, resulting in a more compact and inaccessible form of chromatin, leading to transcriptional silencing. HDAC overexpression is associated with tumors, and its inhibition exhibits a therapeutic effect against different types of solid cancers. Romidepsin was known to inhibit both class I and II HDACs at nanomolar concentrations. This led to increased histone acetylation, ultimately causing significant apoptosis in cancer cell lines derived from CTCL patients [137,138]. The effectiveness of romidepsin was extended towards treating peripheral T-cell lymphoma (PTCL), a heterogeneous group of neoplasms originating in T-cells that is relatively rare and aggressive [139]. Investigators successfully illustrated the synergistic effect between epigenetic drugs, including HDAC and DNA methyl transferase inhibitors, across cell lines and in xenograft models. This preclinical data motivated the need to perform a phase I trial to evaluate romidepsin and 5-azacytidine in patients with PTCL and B-cell lymphoma [190]. In this study, treatment-naïve patients had a higher overall response rate than relapsed/refractory patients. There was a marked reduction in tumor burden, with a median response duration of 20.3 months, lasting longer in treatment-naïve patients. Recently, investigators combined romidepsin with an immunomodulator lenalidomide to assess the efficacy in previously untreated PTCL patients. The phase II study showed a 65.2% overall response rate and 26.1% complete response [191]. Patients with angioimmunoblastic T-cell lymphoma demonstrated a 78.6% overall response rate and 35.7% complete remission. The estimated progression-free survival observed with this study was 48.6% at 1 year and 31.5% at 2 years, with an overall survival of 49–71% [191]. Over the years, CHOP has been established as the frontline treatment for PTCL; however, it demonstrates limited efficacy. The impact of romidepsin on CHOP in the treatment of PTCL was also tested clinically; however, there were negative outcomes from these trials [192,193].

Solid cancers, such as prostate cancer, are known to be prevalent with aberrant epigenetic modifications and have a poor clinical outcome. Androgen deprivation remains a major therapeutic option for this malignance. Ma et al. examined if romidepsin exerts dual effects on both androgen signaling and DNA damage using androgen-sensitive and resistant prostate cancer cell lines [140]. In the study, romidepsin decreased the cell viability of LNCaP and 22Rv1 in a dose-dependent manner. Moreover, gene ontology analysis from RNA-seq data showed that romidepsin was able to suppress pathways associated with prostate development and progression. Anomalous changes in chromatin configuration and DNA methylation are some epigenetic variations that play a key role in carcinogenesis. The combined impact of romidepsin with CC-486, a DNA methyl transferase inhibitor, has been evaluated in advanced solid tumors. Most of the tumor types in this study were CRC. Although partial or complete responses were not attained, stable disease condition was observed in four patients, with one patient maintaining this condition for over 4 months [194]. Thus, the potential clinical application of romidepsin in solid malignancies would be valuable, and further research is warranted to determine if romidepsin and other DNA methyltransferase inhibitors could synergistically induce re-expression of tumor suppressor genes.

#### 3.3.2. Mechanism of Bioactivation

**Figure 8:**
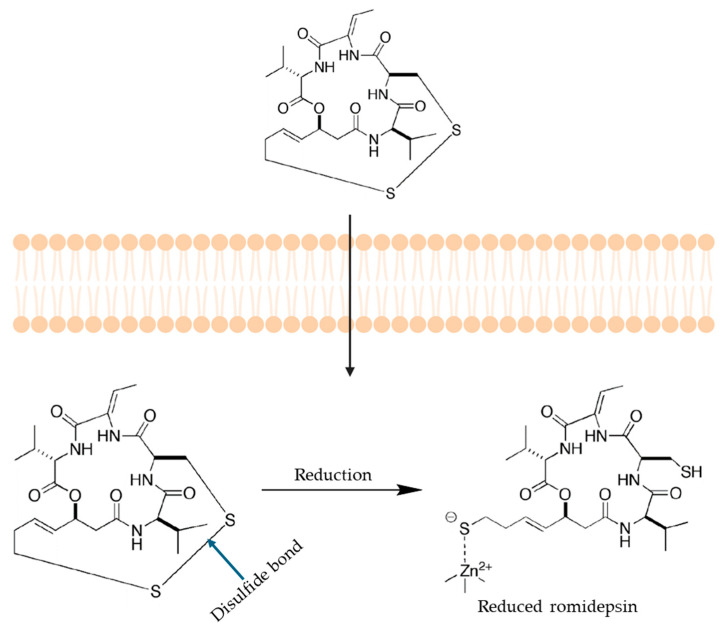
Schematic representation of bioactivation of Romidepsin. Romidepsin, a depsipeptide prodrug is converted to an active inhibitory thiol by in vivo reduction to generate a metabolite which can coordinate the zinc ion in the active site of HDAC.

Romidepsin is a bicyclic peptide containing a disulfide linkage. As represented in Figure 8, upon cell uptake, the disulfide bond is reduced by different cellular reducing agents to generate the reduced form of romidepsin, which displays potent HDAC inhibition [195]. Furumai et al. demonstrated that the reduced form of romidepsin was active, as evidenced by the enhanced IC_50_ for HDAC inhibition by adding reducing agents such as dithiothreitol [196,197]. Glutathione is a tripeptide enzymatically biosynthesized from L-glutamate, L-cysteine, and glycine that plays a key role as a reducing agent intracellularly. In vitro testing using glutathione synthetase mutants in yeast showed that romidepsin suppresses the acetylation of histone H4 in these mutants, indicating that glutathione could be responsible for reducing romidepsin to its active form [196].

The HDAC active site has characteristics of both a metalloprotease and a serine protease. Previous crystallographic studies with another HDAC inhibitor, Trichostatin A (TSA), illustrated that TSA could insert its long aliphatic chain into the tube-like pocket of HDAC. This allows the hydroxamic acid functional group in TSA to coordinate with zinc and other active-site residues through its carbonyl and hydroxyl groups, forming a pentacoordinated zinc complex leading to enzyme inhibition [198]. Without an X-ray crystal structure, it was speculated that the thiol side chain of the reduced form of romidepsin coordinates the zinc ion. A 4-carbon chain spacer connects the sulfhydryl group to the depsipeptide core [196]. The active site residues of HDAC are accessible to the sulfhydryl functional group. Notably, cysteine-151 was identified as a conserved residue in the pocket of all HDAC enzymes. The active form of romidepsin could form a disulfide bond with this cysteine residue. On the other hand, mutating cysteine to serine in HDAC made it less sensitive to romidepsin, although it was not completely ineffective against the mutant HDAC enzyme [189]. As the cysteine mutant of HDAC was not utterly resistant to romidepsin, it could be speculated that the bond between the reduced form of romidepsin and cysteine might not be covalent and that the inhibitory effect might be reversible.

Romidepsin, which is highly protein-bound in plasma and demonstrates reasonable stability, is considerably more hydrophobic than its reduced active form. This characteristic enhances its ability to penetrate cell membranes. Consequently, romidepsin serves as a stable inactive prodrug for its reduced metabolite. Once inside cells, romidepsin is activated to exert its anticancer effects by inhibiting HDAC. This inhibition subsequently reduces chromatin accessibility, thereby silencing the transcription of genes involved in tumor formation.

#### 3.3.3. Off-Target Toxicity

Early evidence of romidepsin’s cardiac toxicity was reported during its preclinical and phase I studies. Electrocardiograph changes, including ST/T wave flattening, ST depression, ST inversion, and QTc prolongation, occur with romidepsin [141]. In a case report, romidepsin was associated with a heart rate increase, asymptomatic arrhythmias, and symptomatic atrial fibrillations [142]. Direct evidence to corroborate this has not yet been established, but acetylation of hERG channels may play a role in QT prolongation. In a phase II single-agent non-randomized study of romidepsin in CTCL or PTCL patients, atrial fibrillations were associated with electrolyte imbalance, including hypomagnesemia and hypokalemia [199]. Elevated troponin levels, a sensitive marker of cardiac muscle, were also reported among these patients. Romidepsin has also been identified as a substrate of ATP-binding cassette transporter ABCB1 (P-glycoprotein, MDR1) [200]. Patients carrying variant alleles of ABCB1 are thought to express higher levels of this efflux transporter in the cardiac endothelium, and these patients have demonstrated a moderately lower risk of developing QT prolongation after romidepsin treatment [143,201]. In addition to cardiac changes, romidepsin can lead to transient elevation of serum alanine transferase and aspartate aminotransferase, but no significant hepatotoxicity was reported [202]. As romidepsin has been shown to cause a mixed cardiac safety profile, caution needs to be taken when prescribing this HDAC inhibitor to patients with pre-existing cardiac conditions and electrolyte imbalance issues.

### 3.4. Other Non-Alkylating Prodrugs

**Figure 9:**
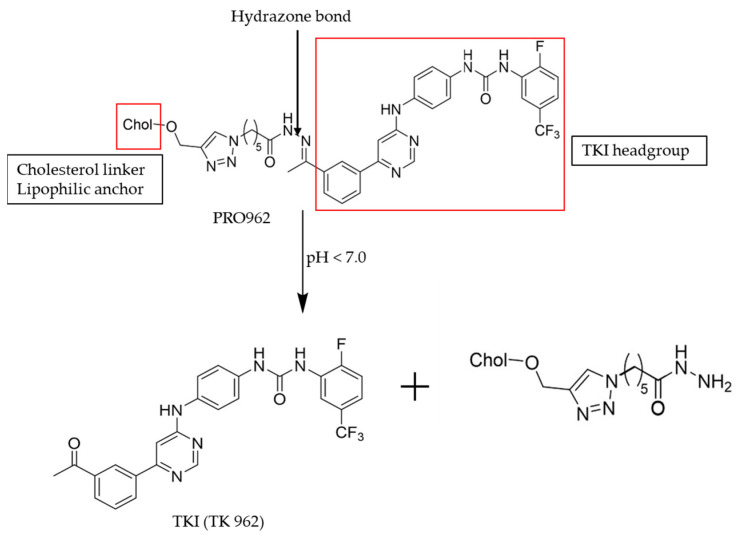
Schematic representation of bioactivation of PRO962. In this prodrug, the tyrosine kinase inhibitor (TKI) is linked to a cholesterol linker through a hydrazone bond. The active compound TK 962 is released when the pH < 7.0, along with the release of the lipophilic anchor. TKI: tyrosine kinase inhibitor.

Among various types of non-alkylating chemotherapeutic drugs, tyrosine kinase inhibitors have been predominantly targeted and have dramatically influenced cancer therapy. However, due to the broad expression of these tyrosine kinases in normal tissues, various off-target effects can occur. Certain drugs are not inherent prodrugs, but their chemical structure can be manipulated to design prodrugs, which can improve their physicochemical properties. TK 962, an investigational tyrosine kinase inhibitor (TKI), was developed into a prodrug to help achieve better stability, bioavailability, and cell internalization [203]. As depicted in Figure 9, TK 962 was attached to a cholesterol moiety through a pH-sensitive hydrazone bond, resulting in a prodrug called PRO962. This prodrug was further examined for its efficacy against pancreatic cancer cell lines in monolayer and 3D models. Conventional liposomal formulation of TK 962 is unstable in human plasma due partly to the non-specific drug release, making it less effective. However, in the case of Lipo2 PRO962, TK 962 was anchored to a liposomal bilayer using cholesterol and a pH-sensitive linker, which enables this prodrug formulation to release the drug intracellularly upon fusion with lysosomes. PRO962 demonstrated effective cytotoxicity in BxPC-3 and A431 (epidermoid carcinoma) bidimensional cells and A431 3D models [203].

**Figure 10:**
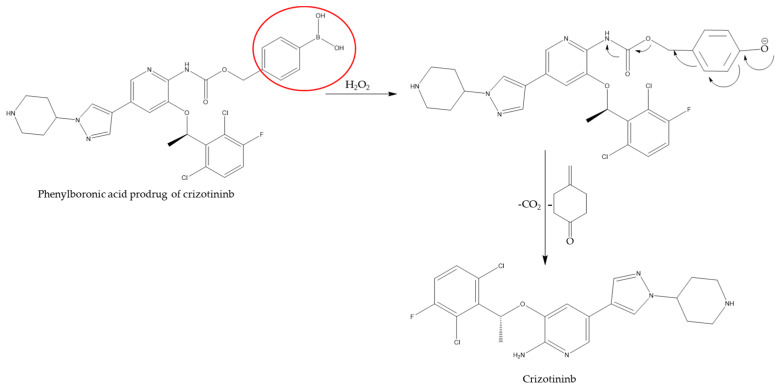
Schematic representation of bioactivation of crizotinib prodrug. In the presence of H_2_O_2_, the phenylboronic acid residue present in this prodrug generates boric acid, which is readily hydrolyzed, and the free drug is released via an electron-migration cascade. H_2_O_2_: hydrogen peroxide.

Crizotinib, a multi-kinase inhibitor, was approved by the FDA for treating anaplastic lymphoma kinase-positive non-small-cell lung cancer in 2011 [204]. Recently, crizotinib was developed into a prodrug to ensure its selective activation within tumor tissue, taking advantage of the increased reactive oxygen levels in these malignant tissues. The carboxylation of the 2-aminopyridine functional group of crizotinib was ideal for prodrug design. The crizotinib prodrug also contains a reactive oxygen species-sensitive phenylboronic acid residue, as seen in Figure 10, which, in the presence of hydrogen peroxide, generates boric acid that is readily hydrolyzed, releasing the parent drug [205]. When the prodrug of crizotinib was tested in different cell lines with varying levels of intracellular reactive oxygen species, the mesenchymal–epithelial-transition-factor-dependent non-small-cell lung cancer cell line H1993 was the only cell type to activate this prodrug, as the concentration of reactive oxygen species is the highest [205]. Similarly, amino ester prodrugs of Bruton’s tyrosine kinase inhibitors have also been developed and investigated [206]. Thus, these strategies can help mitigate the various side effects that typically occur upon using several important classes of anticancer agents.

## 4. Antibody-Drug Conjugates (ADCs)

ADCs are targeted therapeutics developed by conjugating a monoclonal antibody (mAb) to a pharmacologically active payload via a chemical linker [207]. Anticancer ADCs leverage cancer-specific monoclonal antibodies to deliver highly cytotoxic agents directly to malignant cells, while sparing normal tissues. This prodrug design strategy is particularly attractive for cancer therapeutics, leading to extensive research and numerous ADCs currently undergoing clinical studies, with more than 10 ADCs approved by the FDA for clinical oncology treatment [208]. As a heightened research topic, the literature on ADCs is also extensively reviewed [207,209,210,211].

### 4.1. Clinical Significance

Both DNA alkylating and non-alkylating agents can be used as payloads for ADCs. In the past decade, more than 15 duocarmycin-based ADCs have been studied preclinically. One such ADC is SYD985, which was fractionated from SYD983 and developed to target HER2 using the mAb trastuzumab [212]. A phase I dose-escalation and dose-expansion study for SYD985 was conducted between 2014 and 2018, in which 39 patients were enrolled for the dose-escalation study while 146 patients were included and treated for the dose-expansion phase [213]. The HER2-positive patients pretreated with SYD985 had an overall response rate of 33%, with a median progression-free survival of 7.6 months. The overall response was also increased in heavily pretreated patients with low HER2 expression. Additionally, toxins released from the intracellular processing of SYD985 could mediate a bystander effect. These encouraging results enabled SYD985 to be granted fast-track status by the FDA, advancing it to later stages of clinical trials. Triple-negative breast cancer (TNBC) is an aggressive form of breast cancer known to be a therapeutic challenge due partly to the lack of hormonal-targeted treatment. Recently, sacituzumab govitecan (SG), an ADC linking the topoisomerase I inhibitor SN-38 to a humanized hRS7 antibody, exhibited promising outcomes in multiple clinical TNBC treatment trials [214]. Through interaction with trophoblast cell surface antigen 2, the protein target of the hRS7 antibody, SG is internalized into TNBC cells, where SN-38 is released via lysosomal hydrolysis. SN-38, a highly cytotoxic agent, is normally detoxified through glucuronidation by UGT1A1 predominantly in the liver [215]. The ADC approach not only prevents SN-38-induced off-target toxicity but also avoids its quick metabolism and elimination.

### 4.2. Mechanisms of Action

A typical ADC is composed of a mAb, a toxic payload, and a linker. A specific mAb forms the backbone of the ADC. It determines the specificity of the treatment and the location where the payload will be delivered. As such, an ideal antigenic target of a mAb needs to be (1) highly or uniquely expressed in cancer but not normal cells; (2) located on the surface of the cancer cells, which can be easily targeted by the circulating mAb; and (3) such antibody–antigen interaction can trigger endocytosis of the ADC [207]. Cell membrane proteins such as CD-33, CD-22, CD-30, CD-79b, and HER-2 with high preferential expression in malignant cells are commonly used in ADC development [211].

Linkers are another important component of ADCs, including both cleavable and non-cleavable linkers. Cleavable linkers are designed to remove the payload from mAb through enzymatic deconjugation or the tumor microenvironment, such as in hypoxia or acidic conditions [216]. Specifically, hypoxia increases the expression of nitroreductase by several magnitudes in comparison to that in normoxic cells [217]. Taking advantage of this feature, Wang et al. recently developed the first hypoxia-sensitive ADC using a nitroimidazole-based linker, achieving a 27-fold cytotoxicity enhancement in hypoxic cancer cells [218]. Enzymes such as lysosomal protease, β-glucuronidase, sulfatase, and glutathione are broadly used for target-specific payload release. In contrast to cleavable linkers, non-cleavable linkers form stable bonds bridging mAb to payloads; such bonds are usually resistant to enzymatic degradation, and the payload was eventually released post-degradation of the mAb in the lysosomes of tumor cells [219]. One example of this class is the FDA-approved trastuzumab Emtansine (Kadcyla^®^) used for HER2-positive breast cancer. Utilizing a non-cleavable maleimidomethyl cyclohexane-1-carboxylate thioether linker, the ADC only releases the toxic DM1 inside cancer cells after the degradation of trastuzumab [220].

Finally, the success of ADCs requires a highly toxic payload to exert the cancer-killing action. An optimal payload is required to (1) have high water solubility and stability in the systemic circulation; (2) exhibit high cytotoxicity, with in vitro IC_50_ values in the sub-nanomolar concentration ranges; (3) contain chemical moieties that can be conjugated to the linker; and (4) have low molecule weight, low immunogenicity, and a long half-life. Considering all these characteristic requirements, the number of toxic agents that have been successfully used as ADC payloads is limited. Microtubule-disrupting agents and DNA-damaging agents are the primary two classes of compounds often used as anticancer payloads [207].

### 4.3. Off-Target Toxicity

Although designed to enhance target specificity for cancer treatment, most ADCs experience low tumor-specific accumulation, often with less than 1% of the dosage reaching the targeted cells [218] (#). Off-target side toxicity remains a significant safety concern, hindering ADC development. Mechanistically, each of the three components of ADCs can contribute to their side toxicities. For instance, the non-specific expression of targeted antigens in normal cells can mistakenly deliver ADCs to the wrong site. Premature deconjugation of the toxic payload in circulation represents a key mechanism for severe side toxicity associated with the majority of ADCs. Notably, most of the FDA-approved ADCs use cleavable linkers, yet they are more vulnerable to plasma proteases and other enzymes compared to non-cleavable linkers. In contrast, ADCs with non-cleavable linkers may exhibit a favorable safety profile. However, they usually experience low efficacy issues. Thus, future studies developing novel strategies to overcome this unwanted toxicity are warranted.

## 5. Conclusions and Future Perspectives

Prodrugs are a valuable design strategy that has benefited drug development and can be applied to modify conventional chemotherapy. During the period 2012–2022, the FDA granted approval to 50 prodrugs [221]. Several potent molecules that lack the desired formulation properties can be converted into prodrugs. For example, introducing an ionizable moiety converts irinotecan to SN-38, which has much better aqueous solubility. Drugs that undergo extensive first-pass metabolism can be modified by protecting the metabolically labile functional groups through prodrug design. Prodrugs can achieve targeted drug action, making them particularly advantageous in chemotherapy. They can also be developed for site-directed drug delivery by exploiting the predominantly expressed enzymes for their bioconversion. This strategy is beneficial for chemotherapeutic prodrugs, which can be bioactivated by both CYPs and non-CYPs.

Several chemotherapeutic agents widely used in the clinic today are prodrugs, such as CPA, 5-Fluorouracil, and irinotecan. The efficacy of enzymatically bioactivated prodrugs relies directly on the activity of these enzymes. Thus, transcriptional regulation of genes that encode these enzymes can facilitate the generation of therapeutically active metabolites. This can be specifically beneficial in the case of CPA since its bioactivation is primarily mediated by CYP2B6, a highly inducible gene known for extensive inter-individual variability. Enhancing CYP2B6 activation has been explored to augment CPA bioactivation, which can ultimately impact the formation of the therapeutically active metabolite [222,223]. Our laboratory has investigated the effect of CYP2B6 inducers, including CITCO and its analogs, on CPA therapeutic efficacy [224]. Utilizing a lymphoma xenograft mouse model, we were able to illustrate that CITCO pretreatment facilitates CYP2B6-mediated CPA bioactivation, which has a beneficial impact on the anti-neoplastic action of CPA on tumor growth [225].

Gene-directed enzyme prodrug therapy (GDEPT) is an emerging drug design approach where transgenes encoding enzymes required for prodrug activation are delivered to the tumor microenvironment to generate cytotoxicity towards cancer cells [226]. The effectiveness of GDEPT depends on parameters such as the design of the gene-therapy vectors, the chemistry of the prodrug and its active metabolite, and the method of delivering both the gene and the prodrug to the target cells [227]. Enzymes utilized for GDEPT could either be of non-mammalian or human origin, such as viral tyrosine kinase (TK), bacterial cytosine deaminase (CD), or CYP enzymes [228]. It is important to understand that the success of GDEPT also depends on the bystander effect due to the lack of in vivo expression of the foreign enzymes [229]. Notably, while a promising strategy, to date, there are no FDA-approved GDEPT drugs for chemotherapy. As summarized in Section 4, ADCs represent another promising strategy for targeted drug delivery and cancer therapy. Leveraging tumor-specific mAbs, highly toxic payloads, and versatile linkers, novel ADCs with optimal toxicological profiles and therapeutic efficacy are expected to be forthcoming.

Despite all the advantages regarding prodrug design, we need to acknowledge that assessing prodrugs intended for human is challenging. Some enzymes used for prodrug activation or ADC payload release are not selectively expressed in the target cells only or at levels that can easily distinguish them from normal cells. Evaluating the PK profile, drug–drug interactions, and toxicity of the active metabolite following prodrug bioactivation in an animal model may not accurately predict its behavior in humans [1]. Additionally, in the case of prodrugs that require esterase for conversion to the active moiety, significant variation can occur among animal species, and the results may not translate well to human clinical studies. Unwanted side effects remain the predominant concern for all chemotherapeutic agents.

Overall, designing prodrugs has been a successful tool in the drug discovery process because it allows medicinal chemists to develop promising active prototypes that may have undesirable physicochemical properties. Over the years, the use of prodrugs has extended to facilitate the synthesis of molecules that achieve better target selectivity by manipulating conditions distinctively prevalent in tumor cells. This approach is advantageous in chemotherapy because conventional chemotherapeutic agents have been marred by the lack of selectivity, resulting in severe complications among individuals who must endure this treatment for an extended period.

## Figures and Tables

**Table 1:** Alkylating prodrugs and their cancer targets, bioactivation mechanisms, and side toxicities.

Alkylating Prodrug	Target Cancer	Bioactivation Mechanism	Off Target Toxicity
Cyclophosphamide	Non-Hodgkin lymphoma, breast cancer, small-cell lung cancer, graft-vs.-host disease [21,22,23,24,25,26]	CYP2B6	Cardiac tissue injury, nausea, vomiting, diarrhea, hemorrhagic cystitis, and weakening of the immune system [27,28,29,30]
Dacarbazine	Neuroendocrine tumor, malignant melanoma, and Hodgkin lymphoma [31,32,33,34]	CYP1A2	Hepatotoxicity, leukopenia, thrombocytopenia, drop in blood pressure, and severe acute sinusoidal obstruction syndrome [35,36,37]
Duocarmycin	Colorectal cancers [38,39]	CYP1A1, CYP2W1	Hepatotoxicity, myelosuppression, fatigue, conjunctivitis, hyperpigmentation, and erythema [40,41,42]
Evofosfamide	Glioblastoma [43]	Hypoxia	Thrombocytopenia, hyperpigmentation, and skin ulceration [44]
